# Genome-wide expression analysis in a Fabry disease human podocyte cell line

**DOI:** 10.1016/j.heliyon.2024.e34357

**Published:** 2024-07-09

**Authors:** Sarah Snanoudj, Céline Derambure, Cheng Zhang, Nguyen Thi Hai Yen, Céline Lesueur, Sophie Coutant, Lénaïg Abily-Donval, Stéphane Marret, Hong Yang, Adil Mardinoglu, Soumeya Bekri, Abdellah Tebani

**Affiliations:** a Normandie Univ, UNIROUEN, INSERM, U1245, CHU Rouen, Department of Metabolic Biochemistry, Referral Center for Lysosomal Diseases, Filière G2M, 76000, Rouen, France; b Normandie Univ, UNIROUEN, INSERM U1245 and CHU Rouen, Department of Genetics and Reference Center for Developmental Disorders, FHU-G4 Génomique, F-76000, Rouen, France; c Science for Life Laboratory, KTH - Royal Institute of Technology, Stockholm, Sweden; d Normandie Univ, UNIROUEN, INSERM, U1245, CHU Rouen, Department of Neonatal Pediatrics, Intensive Care, and Neuropediatrics, 76000, Rouen, France; e Centre for Host-Microbiome Interactions, Faculty of Dentistry, Oral & Craniofacial Sciences, King's College London, London, United Kingdom

**Keywords:** Fabry disease, RNAseq, Transcriptomics, Metabolic modeling, Systems biology, Podocyte

## Abstract

Fabry disease (FD) is an X-linked lysosomal disease caused by an enzyme deficiency of alpha-galactosidase A (α-gal A). This deficiency leads to the accumulation of glycosphingolipids in lysosomes, resulting in a range of clinical symptoms. The complex pathogenesis of FD involves lysosomal dysfunction, altered autophagy, and mitochondrial abnormalities. Omics sciences, particularly transcriptomic analysis, comprehensively understand molecular mechanisms underlying diseases. This study focuses on genome-wide expression analysis in an FD human podocyte model to gain insights into the underlying mechanisms of podocyte dysfunction. Human control and GLA-edited podocytes were used. Gene expression data was generated using RNA-seq analysis, and differentially expressed genes were identified using DESeq2. Principal component analysis and Spearman correlation have explored gene expression trends. Functional enrichment and Reporter metabolite analyses were conducted to identify significantly affected metabolites and metabolic pathways. Differential expression analysis revealed 247 genes with altered expression levels in GLA-edited podocytes compared to control podocytes. Among these genes, 136 were underexpressed, and 111 were overexpressed in GLA-edited cells. Functional analysis of differentially expressed genes showed their involvement in various pathways related to oxidative stress, inflammation, fatty acid metabolism, collagen and extracellular matrix homeostasis, kidney injury, apoptosis, autophagy, and cellular stress response. The study provides insights into molecular mechanisms underlying Fabry podocyte dysfunction. Integrating transcriptomics data with genome-scale metabolic modeling further unveiled metabolic alterations in GLA-edited podocytes. This comprehensive approach contributes to a better understanding of Fabry disease and may lead to identifying new biomarkers and therapeutic targets for this rare lysosomal disorder.

## Highlights

• Fabry disease is an X-linked lysosomal disease related to glycosphingolipids catabolism impairment.
• Fabry disease's broader molecular mechanisms were revealed using a human podocyte model and genome-wide expression data.
• Fabry disease pathogenesis involves inflammation, oxidative stress, apoptosis, and autophagy.

## 1 Introduction

Fabry disease (FD, OMIM 301500) is an X-linked lysosomal disease (LD) caused by a deficiency of alpha-galactosidase A (α-d-galactoside galactohydrolase, EC 3.2.1.22; α-gal A) encoded by the *GLA* gene. This enzyme is involved in the degradation of glycosphingolipids [1]. Glycosphingolipids are essential constituents of cell membranes and are widely distributed in human tissues [2]. α-gal A deficiency results in the progressive and systemic accumulation of globotriaosylceramide (Gb_3_ or GL-3) and other related glycosphingolipids in lysosomes [3]. The emergence of new multifaceted roles of the lysosome in several cellular processes and its primary function of macromolecule degradation sheds light on the mechanisms underlying FD and LDs in general [4]. Thus, lysosomes participate in vital processes such as antigen presentation, cell adhesion, apoptotic cell death, interorganellar communication, and gene regulation [5]. In addition, lysosomes are highly mobile within the cell enabling the interaction with various cellular compartments and maintaining cellular homeostasis [6]. The diverse range of functions and interactions introduces a layer of biological complexity to these organelles [5]. A continuum of clinical severity is observed in FD, with the molecular abnormality and the sex of the patient being essential determinants [7]. The severity range spans from a severe classical phenotype to moderate nonclassical phenotype [8]. The classical phenotype is mostly seen in men. Symptoms encompass neuropathic pain, cornea verticillata, angiokeratoma, gastrointestinal involvement, sweating anomalies, hearing loss, hypertrophic cardiomyopathy, cardiac rhythm disorders, progressive renal failure, and stroke, with an onset in infancy [9,10]. The nonclassical phenotype is milder with a later onset, a more variable course of the disease, and can be limited to one or a small number of organs [7]. Despite the X-linked inheritance pattern, women often have signs and symptoms of FD related to random and/or skewed X inactivation, but are usually less severely affected compared to men [11]. Reported incidences range from 1 in 476,000 [12] to 1 in 117,000 [13] in the general population but may be largely underestimated as shown by newborn screening programs with prevalences as high as 1 in 3100 newborns in Italy [14] and 1 in 1500 newborn males in Taiwan [15].

Diagnosis of Fabry disease relies on a deficient α-gal A activity in plasma, leukocytes [16] or dried blood spot [17] in males, confirmed by genotyping. Affected women can exhibit an enzyme activity within the normal range and diagnosis rests on molecular analysis of the *GLA* gene [18]. Available treatment options are enzyme replacement therapy (ERT: agalsidase beta, Fabrazyme®; agalsidase alfa, Replagal® [19]), oral chaperone therapy (Migalastat, Galafold® [20]), and conventional medical treatment to manage the morbidities related to the disease. Phenotype-genotype correlation is partial in FD and the relationship between genotype and quantitative difference levels of substrates in tissues is still not well understood, thus causing different effects on organ changes [21]. Studies have shown that lyso-Gb3 (globotriaosylsphingosine), converted from Gb3 by acid ceramidase within tissues, is a better biomarker than Gb3. It is the only available specific biomarker of FD [22] and is primarily used in the context of initial FD diagnosis. Plasma lyso-Gb3 levels have also been partially correlated with disease severity [23,24]. However, it has not been validated for treatment monitoring and has been shown to not correlate with disease progression or prognosis in treated patients [25]. New specific biomarkers are therefore needed to better classify and monitor Fabry patients. FD pathogenesis is complex and encompasses multiple pathological mechanisms, including but not limited to lysosomal dysfunction, altered autophagy and mitochondrial abnormalities [26]. Better understanding of the pathways involved could result in improved prognosis and yield new biomarkers and therapeutic targets.

The rise of omics sciences has prompted a paradigm shift, in both research and medicine [[27], [28], [29]]. These approaches may bring new insights into the pathophysiology of rare [30,31] and multifactorial complex diseases [32]. Among omics sciences, transcriptomic analysis offers a global picture and may enable the exploration of the molecular mechanisms underlying diseases [33,34]. Kidneys are a central organ target in FD and podocytes, which are a key cellular type in Fabry-related nephropathy, with significant Gb3 and lyso-Gb3 accumulation, glycolipid deposits, and early signs of injury [35]. This study aims to perform genome-wide expression analysis in an FD human podocyte model compared to control podocytes to gain insight into the underlying mechanisms of podocyte dysfunction.

## 2 Material and methods

### 2.1 Podocyte culture

Our study used human immortalized podocytes kindly provided by Dr. Weisz's team [36]. The immortalized human podocytes were collected from a 1-month-old normal woman's kidney. Half of the podocytes (n = 9) were then edited using CRISPR/Cas9 technology targeting the first and the seventh exons of *GLA* on both X chromosomes. The other (n = 9) were used as controls. Podocytes were utilized for CRISPR/Cas9 method at passage number of 9 from the primarily immortalized podocytes. Two groups of cells were numbered: control podocytes (C1 to C9) and CRISPR/Cas9 modified podocytes (F1 to F9). The cells were incubated at 37 °C with 5 % CO2 in Dulbecco's modified Eagles Medium: Nutrient Mixture F-12 or DMEM/F-12 (PAN BIOTECH, P04-41450) supplemented with 10 % fetal bovine serum – FBS (Eurobio, CVFSVF00-01), 1 % glutamine 200 nM (Eurobio, CSTGLU00-0U), 1 % penicillin 100 UI/ml and streptomycin 100 μg/ml (Sigma-Aldrich, P0781), and 0.1 % ITS cell culture supplement (PAN BIOTECH, P07-03110). Medium was exchanged every 2–3 days. Trypsin was used to detach adherent cells, then was deactivated using culture medium. Cells were seeded at a density of 10,000 cells per cm^2^ in fresh cell culture dishes. After 72 h of incubation, the cells were harvested by centrifugation. Cell pellets and supernatants were frozen at −80 °C. HPLC Plus grade water was added, and the cell pellets were sonicated for further analysis.

### 2.2 Gb3 assessment

Gb3 measurement was implemented following to Mills’ method [37]. Cultured cells were scraped with phosphate-buffered saline and centrifuged 10 min at 2000 RPM. The pellet was hydrated using 500 μl of water (Water Plus, Carlo Erba) and mixed using ultrasonic sound 5 times for 2 s. Three hundred μL of this mix were added to 300 μl of water (Water Plus, Carlo Erba) and 50 μl of C17-CTH (Matreya) at 0.05 μg/ml used as calibrator. A sample of 100 μl was used for protein assessment. Extraction was realized with the addition of 5 mL of chloroform (Merck): methanol (Carlo Erba) (2:1, v/v) by shaking on a multivortexer for 10 min. The two layers were separated by centrifugation for 5 min at 3500 RPM. The upper layer was transferred to a new vial and dried under N2 at 40 °C. An amount of 100 μl of methanol was added prior to mass spectrometer analysis with Sciex 4000 QTRAP (AB Sciex) using the electrospray ion source (TurbolonSpray). Ten μL were directly infused into the electrospray via HPLC line (Shimadzu) in flow injection analysis mode.

### 2.3 Alpha-galactocerebrosidase activity analysis

Alpha-galactocerebrosidase enzymatic activity was measured in cell pellets using a fluorometric assay [38]. Briefly, a fluorescent substrate, 4-methylumbellipheryl-α-*d*-galactoside (Sigma-Aldrich), was used. Incubation was carried out in an incubator with shaking, and fluorescence readings were carried out using a fluorometer equipped with a plate reader (SpectraMax Gemini XPS, Molecular Devices) at 365 nm (excitation) and 450 nm (emission). The result is expressed as microkatal per mg of protein (ukatal/mg protein).

### 2.4 RNA extraction and RNA-seq analysis

RNA samples from both cell lines were sequenced at the Rouen University Hospital genomic platform, Rouen, France. Total RNA from podocytes was extracted using NucleoSpin® RNA/Protein kit with a DNase treatment step to avoid contamination with genomic DNA. The quality and quantity of RNA were assessed using a fluorimetric assay on Qbit (ThermoFischer Scientific, Waltham, MA, USA). Libraries were prepared using the NEBNext Ultra II Directional RNA Library Kit with polyA RNA selection (New England Biolabs, Ipswich, MA, USA). One μg of total RNA was used. High-throughput sequencing of the libraries was performed on an Illumina NextSeq 500 (Illumina, San Diego, CA, USA) using 2*75 bp sequencing to generate 30 M read pairs on average per sample. Bioinformatics analysis was carried out using nf-core/RNA-seq v3.1 analysis pipeline to generate multi quality control report that uses the STAR v2.6.1d for alignment [39]. The ouput files are then used as input for FeatureCounts which is used for gene quantification (Ensemble GTF: Homo_sapiens.GRCh37.75.gtf). No quality check at FASTQ level were performed. Visual exploration of the BAM files was performed with the IGV tool from the Broad Institute.

### 2.5 Data analysis

Transcript expression levels were calculated as both raw counts or as transcript per million (TPM). Gene expression levels were calculated by summing up all the TPM values of all alternatively spliced protein coding transcripts of the corresponding gene for a total number of 19,670 protein-coding genes. The average TPM values are used to estimate the gene expression level. All TPM values were TMM normalized [40] between all the samples. Expression level cut-off is set at 1 TPM. A total number of 11798 genes are expressed at 1 TPM or higher in all samples. The full TPM data matrix is shown in Supplementary Table S1. Differential expression analysis was conducted by using mRNA raw counts. The DESeq2 R package [41] was used for differential analysis. Genes that had an adjusted p-value less than 0.05 with the "Benjamini-Hochberg" method were identified as differentially expressed genes. Data analysis and visualization was performed using on R (version 4.0.0) [42]. Clustering in heatmaps and dendrograms based on Spearman correlation were created by first calculating a correlation matrix of Spearman's ρ [43]. The correlation was converted to a distance metric (1 – ρ) and was clustered using unsupervised top-down hierarchical clustering. Dendrograms showing gene expression in heatmaps have been clustered using the Ward2 algorithm an implementation of Ward's minimum variance method [44] implemented as “Ward.D2” in the hclust function in the R package stats. Principal Component Analysis has been performed on log transformed values (log(TPM + 1)) using the R package pcaMethods [45]. Wilcoxon–Mann–Whitney test was applied to examine the statistical difference in total Gb3 level and α-gal A activity between CRISPR/Cas9-modified and control immortalized human podocytes.

### 2.6 Reporter metabolite and subnetwork analysis

A reporter metabolite analysis [46] was performed based on the differential analysis results between Fabry and control cell lines. In brief, this method is used to identify significantly affected metabolites based on the significance of gene changes and topology of genome-scale metabolic models (GEMs). The differential gene expression results were obtained as described above, and the generic human metabolic model generated was used as input for reporter metabolite analysis. The reporter subnetwork was also retrieved using the previously published method, removing 20 currency metabolites connected to too many reactions. The deleted currency metabolites were “H_2_O”, “CO_2_”, “O_2_”, “H^+^”, “HCO_3_^−^”, “Na^+^”, “CoA”, “Pi”, “PPi”, “AMP”, “ADP”, “ATP”, “NAD^+^”, “NADH”, “NADP^+^”, “NADPH”, “PAP”, “PAPS”, “FAD”, and “FADH_2_.”

## 3 Results

### 3.1 Validation of podocyte model generated by CRISPR/Cas9 technology

The mass spectrometry-based analysis exhibited that the total Gb3 level was significantly higher in CRISPR/Cas9-modified podocytes compared to the controls (Wilcoxon–Mann–Whitney test, p-value = 5.74x10^−4^) (Supplementary Fig. 1). The enzyme activity examination using a fluorometric assay showed that almost no α-gal A activity was observed in GLA-edited podocytes with a median of 0.44 μkat/kg while the figure for controls was 18.43 μkat/kg (Wilcoxon–Mann–Whitney test, p-value = 4.01x10^−4^) (Supplementary Fig. 1). These results indicated that the CRISPR/Cas9-edited podocytes were valuable in presenting the genotype and phenotype of Fabry disease.

### 3.2 Differential expression analysis

**Fig. 1:**
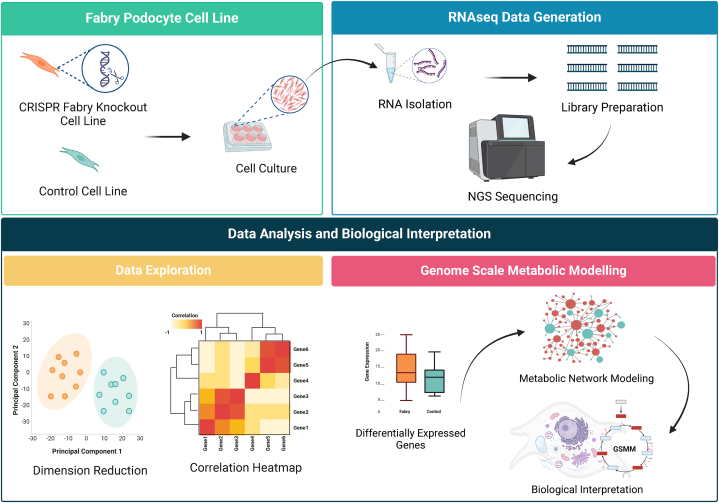
**Overview of the experimental design, data generation, and data analysis.** Abbreviation: NGS, Next-Generation Sequencing.

**Fig. 2:**
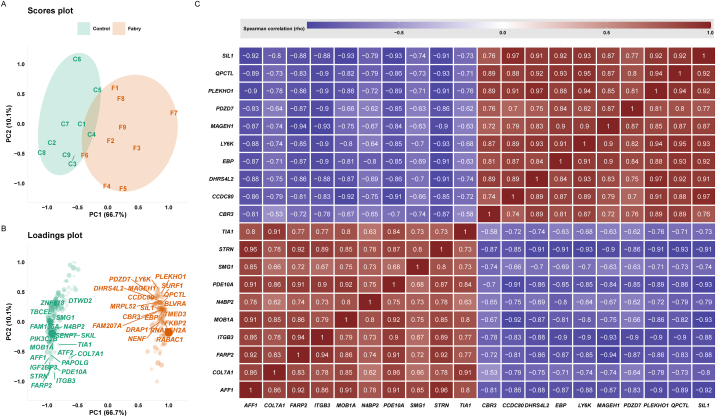
**RNAseq data exploration.** A) Principal component analysis scores plot highlighting the differences between the two groups (n = 9). B) Principal component analysis loadings plot highlighting the top driving gene expression between the two groups. C) Spearman correlation heatmap of the top 20 differentially expressed genes.

This work uses RNAseq data to explore genome expression profile differences between GLA-edited and control podocytes. The study design is depicted in Fig. 1. In total 11798 genes (60 % of all putative protein-coding genes) were expressed in all analyzed samples. The whole data matrix with sample characteristics is presented in Supplementary Table S1. To examine the data, we initially compared two groups; Fabry samples versus control samples. Our analysis revealed 247 genes that showed differential expression (with an adjusted p-value of less than 0.05), of which 136 genes were underexpressed and 111 genes were overexpressed in GLA-edited cells compared to control cells. Expressed genes were sorted according to the fold change. The complete list of genes and their related statistics are presented in Supplementary Table S2. The 10 most overexpressed genes were *MAGEH1, DHRS4L2, LY6K, CBR3, CCDC80, EBP, PLEKHO1, QPCTL, PDZD7,* and *SIL1*. The 10 most underexpressed genes were *COL7A1, PDE10A, ITGB3, N4BP2, AFF1, STRN, TIA1, MOB1A, SMG1,* and *FARP2* (Fig. 2A, Supplementary Table S2). We used a principal component analysis to track how the samples cluster based on their differential expression profiles. Fig. 2A shows the visualization of this data, while Fig. 2B displays the covariation of genes in their related groups. The separation of samples was mostly observed on the PC1 dimension, which alone explains 66.7 % of the dataset variance. The PCA scores’ matrices are presented in Supplementary Table S3. To visualize the covariation of the top genes, we performed a Spearman correlation analysis between the differentially expressed genes. We present in Fig. 2C a heatmap of the correlation between the top 20 genes that clearly shows two clusters, upregulated and downregulated. The total correlation matrix is presented in Supplementary Table S4.

**Fig. 3:**
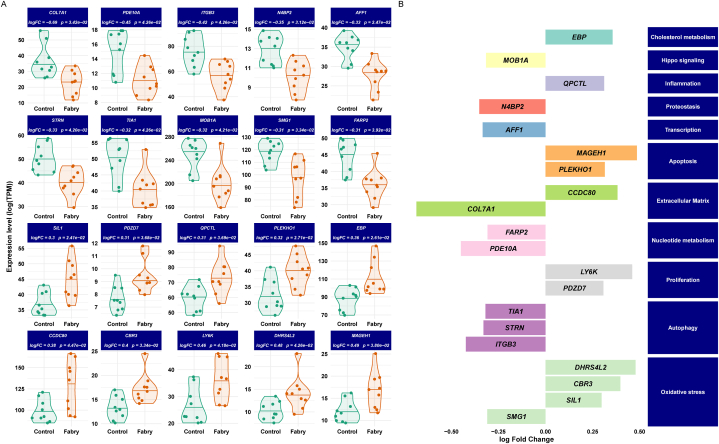
**Differentially expression analysis results.** A) Boxplots of the 20 top varying genes (upregulated and downregulated) between GLA-edited and control podocytes. Lines in the violin plots refer to quantiles (0.25, 050, and 0.75). B) Barplots of the change of the top genes and their functional classification.

To visualize the expression trends, we generated violin plots to show the top 10 upregulated and 10 downregulated genes' expression levels (Fig. 3A). In addition, Fig. 3B shows the functional analysis of the top differentially expressed genes that are mainly involved in the following pathways: Oxidative stress (*CBR3, DHRS4L2, SIL1, SMG1, TIA1*), NAD/NADP-dependent oxidoreductase (*CBR3, DHRS4L2*), inflammation (*CBR3, ITGB3, QPCTL, SMG1*), fatty acid metabolism (*CBR3, CCDC80, DHRS4L2*), collagen and extracellular matrix homeostasis (*CCDC80, COL7A1, ITGB3*), Wnt signaling pathway (*CCDC80, STRN*), kidney injury (*COL7A1, ITGB3, MAGEH1*), apoptosis (*EBP, MAGEH1, PLEKHO1, SMG1, TIA1*), Hippo signaling pathway (*ITGB3, MOB1A, PLEKHO1, STRN*), autophagy *(ITGB3, SIL1, STRN, TIA1*), and endoplasmic reticulum (ER) stress (*SIL1*).

### 3.3 Generation of a genome-scale metabolic network

**Fig. 4:**
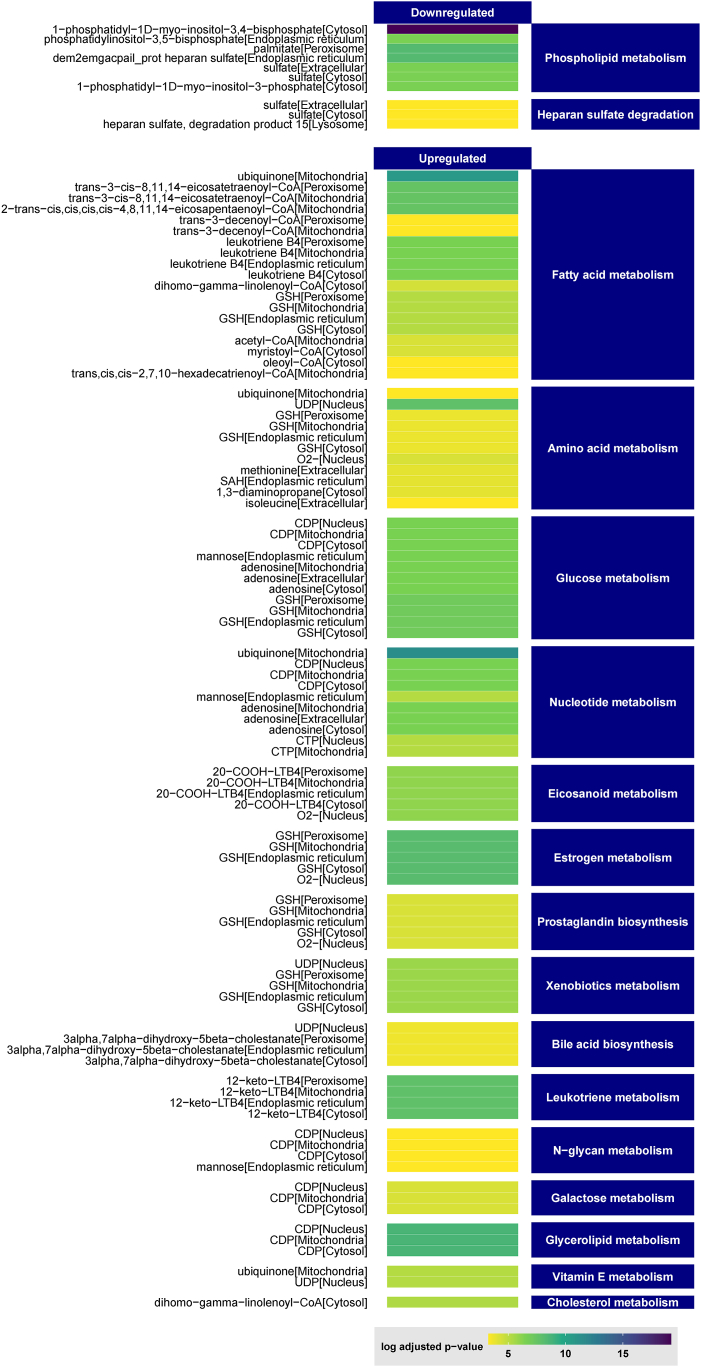
Heatmap of the top reporter metabolites and their metabolic classes predicted by the metabolic modeling.

To investigate the comprehensive dynamics of metabolites in Fabry disease, we used the transcriptomic data to generate a genome-scale metabolic network by integrating all known human metabolic equations and pathways [47]. The generated GEM showed a deep change in metabolic flux with 479 significantly affected reporter metabolites. Sixty-four were downregulated and 419 were upregulated (Fig. 4) and Supplementary Table S5. Functional enrichment analyses showed that phospholipid metabolism and heparan sulfate degradation were downregulated. In contrast, fatty acid metabolism, amino acid metabolism, glucose metabolism, nucleotide metabolism, eicosanoid metabolism, estrogen metabolism, prostaglandin biosynthesis, glutathione metabolism, and leukotriene metabolism were upregulated. The full list is presented in Supplementary Table S6.

## 4 Discussion

**Fig. 5:**
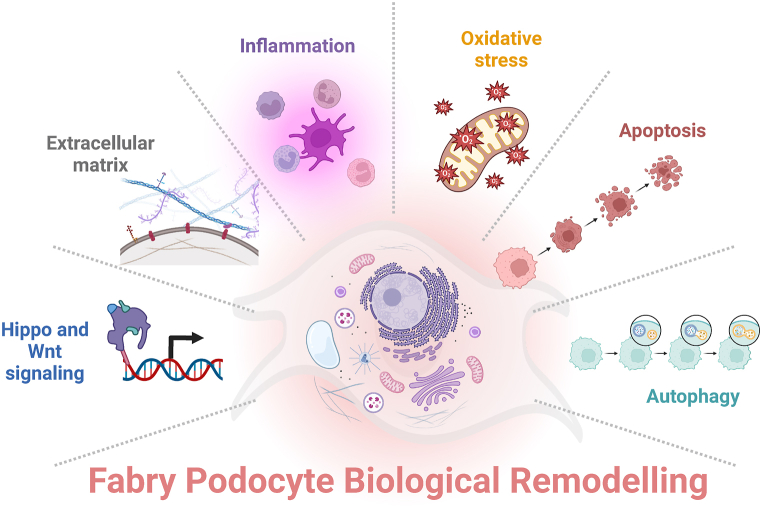
Summary of the biological and metabolic remodeling of GLA-edited podocytes.

Investigating Fabry disease is crucial for early diagnosis, understanding disease mechanisms, and developing effective treatments. Blending high-throughput omics data as transcriptomics and genome-scale metabolic modeling using a podocyte cell line offers a powerful and appropriate approach to uncover key molecular changes associated with Fabry disease and advance our knowledge of this disorder. By applying computational modeling techniques to gene expression data, we can generate comprehensive depictions of biological systems and glean valuable insights into the regulation and functioning of cellular processes [48,49]. To better reflect the dynamic nature of cellular processes, models can be fine-tuned by incorporating transcriptomics data. This type of data, obtained through RNA sequencing, provides information on the gene expression patterns of an organism and offers a snapshot of the genes actively expressed in a specific cell type or tissue. When combined with GEMs, transcriptomics data enables the inference of metabolic pathway activity and a deeper understanding of cellular function in a given condition. The comparison between GLA-edited and control samples identified 247 (136 downregulated and 111 upregulated) genes in CRISPR/Cas9 modified cells. By assessing the functional analysis of the top 10 overexpressed genes (*MAGEH1, DHRS4L2, LY6K, CBR3, CCDC80, EBP, PLEKHO1, QPCTL, PDZD7, and SIL1*) and the top 10 underexpressed genes (*COL7A1, PDE10A, ITGB3, N4BP2, AFF1, STRN, TIA1, MOB1A, SMG1, and FARP2*), it was found that GLA-edited podocytes were most affected in the following biological pathways: oxidative stress, inflammation, collagen and extracellular matrix (ECM) homeostasis, apoptosis, autophagy and Hippo and Wnt signaling. The overall biological remodeling revealed in GLA-edited cells is presented in Fig. 5. It is worth noting that some of these genes are involved in multiple processes.

Oxidative stress is believed to play a crucial role in the pathophysiology of Fabry disease [50,51]. The study identified several genes, *CBR3*, *DHRS4L2*, *SIL1*, *SMG1*, and *TIA1*, previously associated with oxidative stress. *TIA1*, for instance, is an RNA-binding protein that plays a role in apoptotic processes. Binding to particular RNA sequences contributes to forming stress granules that help regulate cell survival and apoptosis [52]. *TIA1* has also been connected to the autophagy [53]. *CBR3*, also known as Carbonyl Reductase 3, is an enzyme that helps detoxify carbonyl compounds. Its main function is to break down reactive carbonyl species produced during oxidative stress, which helps mitigate its effects. CBR3 is involved in various metabolic pathways such as leukotriene metabolism, prostaglandin biosynthesis, arachidonic acid metabolism, omega-3 fatty acid metabolism, eicosanoid metabolism, and xenobiotics metabolism [54]. *DHRS4L2* is a part of the SDR family, which stands for short-chain dehydrogenase/reductase. Members of this family play a role in protecting cells against oxidative stress by participating in cellular antioxidant mechanisms. The *SIL1* gene, which produces the Nucleotide Exchange Factor SIL1, plays an important role in regulating the folding of proteins in the ER. When exposed to oxidative stress, protein folding can become disrupted, leading to the buildup of misfolded proteins and ER stress [55]. The *SMG1* gene, or Suppressor of Morphogenesis in Genitalia 1, is crucial in the degradation of mRNA that contains nonsense mutations. It is also involved in pathways related to genotoxic and oxidative stress [56,57]. *SMG1* expression is downregulated during the inflammatory process [58].

In Fabry disease, inflammation is also an important factor to consider. Chronic inflammation can be caused by Fabry disease, as there is a buildup of unprocessed glycolipid substrates. This inflammation is triggered by pathogenic cascades [59]. Furthermore, Fabry disease can impact the immune system and cause an over-expression of immune molecules, leading to leukocyte perturbation. The activation of innate immunity can be initiated by dendritic cells through toll-like receptors and Gb3 and/or lyso-Gb3. This can lead to inflammation and fibrosis [60]. When Gb3 accumulates in the kidneys, it can increase pro-fibrotic molecules and result in renal fibrosis [61]. Several top highlighted genes in this work, including *CBR3, ITGB3, SMG1*, and *QPCTL*, are also associated with inflammation. One example is *ITGB3* (Integrin Beta 3), which encodes the integrin beta-3 subunit. Integrins facilitate communication between immune cells and the extracellular matrix during the inflammatory response. Additionally, ITGB3 is involved in autophagy by regulating the formation of autophagosomes and related signaling pathways [62]. Another gene is *QPCTL* (Glutaminyl-peptide cyclotransferase-like protein) encodes a protein called glutaminyl-peptide cyclotransferase-like protein, which is involved in post-translational modifications. *QPCTL* is upregulated in macrophages during inflammation and may participate in modulating the immune response [63].

The ECM is essential for maintaining the structural integrity of cells and provides support and stability. Additionally, it regulates cell signaling, migration, and proliferation [64]. Interestingly, this work has identified several ECM-related genes (*CCDC80*, *COL7A1*, *ITGB3*) that help maintain ECM homeostasis and integrity, ensuring proper structure and function. Among these genes*, CCDC80* (Coiled-Coil Domain Containing 80) is involved in ECM remodeling by contributing to collagen fibers stabilization and organization [65]. *COL7A1* (Collagen Type VII Alpha 1 Chain) is a major component of the anchoring fibrils within the dermal-epidermal junction. Type VII collagen is localized in the basement zone beneath stratified squamous epithelia and is an anchoring fibril between the external epithelia and the underlying stroma [66]. Alterations in the composition and organization of the ECM have been described in FD [67]. Fibrosis can occur early in target organs such as the heart and the kidney [67]. In the kidney, the glomerular basement membrane, a specialized ECM essential for kidney function, is altered in Fabry disease [68]. Gb3 accumulation disrupts the composition and organization of the glomerular basement membrane, leading to thickening, expansion and fibrosis [69]. Moreover, lyso-Gb3 activates Notch1 signaling, activating NFκB and transcribing proinflammatory and ECM genes [70]. These changes disrupt renal filtration and contribute to the development of proteinuria and renal dysfunction. Besides, some integrins have been involved in podocyte detachment from the glomerular basement membrane leading to podocyturia. Interestingly, the increase in *ITGB3* expression reported in this study is supported by the elevated urinary ITGB3 concentration in Fabry patients compared to the control group reported by Utsimi et al. [71].

The process of programmed cell death, known as apoptosis, occurs naturally in the body to eliminate damaged or unnecessary cells. This work has identified several genes, such as *EBP, MAGEH1, PLEKHO1, SMG1*, and *TIA1*, that are reported to be involved in apoptosis through various mechanisms. *MAGEH1,* a member of the *MAGE* gene family, is involved in causing caspase-dependent apoptosis of renal tubular cells through the use of nephrotoxic drugs [72]. Studies have shown that PLEKHO1, a member of the Pleckstrin homology domain-containing family, is involved in regulating apoptosis. When PLEKHO1 is overexpressed, it promotes apoptosis [73]. A higher apoptotic rate has been observed in patients with FD and is directly attributed to elevated Gb3 levels [74]. This rate is reduced in treated patients compared to untreated ones [74,75]. Importantly, it has been demonstrated that podocyte injury and loss in Fabry disease is, at least, partially related to an increased apoptotic rate [76].

Autophagy, a process maintaining cellular homeostasis and preventing harmful material accumulation was also revealed. In Fabry disease, disruption of autophagic flux has been demonstrated, with altered autophagosome maturation contributing to the pathophysiology of the disease [77]. Additionally, there is a loss of mTOR kinase activity [78], which further affects the regulation of autophagy. Autophagy disruption may lead to the accumulation of misfolded proteins and damaged organelles within podocytes [79]. Consequently, this impairment may contribute to podocyte injury and the subsequent proteinuria, hallmark of kidney dysfunction [78]. Among the top upregulated genes, *SIL1* (Silver homolog) is primarily known for its role in protein folding and ER homeostasis. However, recent studies have implicated *SIL1* in regulating autophagy and the clearance of misfolded proteins through the ER-associated degradation (ERAD) pathway [80].

Hippo and Wnt signaling are two important pathways biology that are highlighted in this study [81,82]. Hippo signaling pathway plays a significant role in controlling organ size and regulating cell proliferation, apoptosis, and differentiation [82]. On the other hand, the Wnt signaling pathway is involved in controlling embryonic development, cell growth, and differentiation [81]. Among the top differentially expressed genes, *STRN* (striatin) and *MOB1A* interact in the hippo pathway [83]. *STRN* is involved in regulation of signal transduction pathways, mitosis and cytokinesis, cell polarity, autophagy, and protein trafficking [84,85]. *STRN* has also been reported as a component of the mTORC1 signaling pathway, a crucial regulator of autophagy [85]. Moreover, the hippo signaling is interconnected with the Wnt signaling pathway and regulates both innate and adaptive immunity [86]. A recent study reported that a hyper-activation of hippo pathway alters the growth/death balance and may underlie the neuronal loss in neuronopathic Gaucher disease [87], which suggested the association between hippo pathway and neurological pain in FD [88]. In addition, other major partners in Hippo signaling are under-expressed in Fabry podocytes, such as *LATS1* and *YAP1*. Further research is needed to explore the potential involvement of Hippo signaling and its downstream effectors in Fabry disease.

From a metabolic modeling perspective, this study revealed a striking transformation in the metabolic flux, exerting a substantial influence on 479 reporter metabolites in which 64 displayed downregulation trends. The analysis showed changes in phospholipid metabolism, which could indicate a change in the composition or integrity of the cell membrane. Additionally, the study revealed a decrease in heparan sulfate degradation, an important component of the extracellular matrix that goes through lysosomal degradation. This suggests potential changes in cellular interactions with the ECM, which aligns with the remodeling of ECM as mentioned above. Furthermore, there was an increase in activity observed in several metabolic pathways, including fatty acid metabolism, amino acid metabolism, glucose metabolism, nucleotide metabolism, eicosanoid metabolism, estrogen metabolism, prostaglandin biosynthesis, glutathione metabolism, and leukotriene metabolism. These results suggest that the cells utilize and allocate energy differently, producing more key molecules and potentially altering signaling processes. Fatty acid and glucose metabolism are crucial for energy production, while amino acid and nucleotide metabolism are necessary for protein synthesis and DNA/RNA production which also resonates with above-mentioned remodeling of oxidative stress and inflammation. Besides, alterations in metabolic flux, cellular energy allocation, and macromolecule degradation were observed.

Overall, these results provide valuable insights into the metabolic rewiring that occurs in Fabry disease. By combining transcriptomics and genome-scale metabolic modeling, this study of Fabry disease has shed light on crucial molecular transformations underlying the disorder as well as the potential consequences and functional adaptations in GLA-edited podocytes. Regarding using the human podocyte model, Fabian et al. focused on the transcriptome and proteome alterations in response to ERT, revealing α-synuclein storage as a crucial factor mediating podocyte injury [89]. Also, Ester et al. applied a high-throughput antibody array in exploring the change in FD at the protein level and phosphorylation [90]. Remarkedly, José et al. concentrated on the proteome alterations in FD, resulting in downregulated proteins associated with tubulointerstitial fibrosis and autophagy [91]. Ulrich's team found dysregulated proteins in α-GAL-deficient podocytes, related to thermogenesis, lysosomal trafficking and function, metabolic activity, cell-cell interactions and cell cycle [92]. In accordance to our previous work investigating proteome profiles of Fabry patients and healthy controls [93], SMAD5 and KITLG were found to be down-regulated while MAGED1, FXYD5, SERPINB6, MVK, UBAC1, GPKOW, PPIB, MESD, TACC3 were up-regulated in plasma Fabry samples compared to controls. Furthermore, our metabolomics investigation on plasma Fabry patients and controls also exhibited the augmentation of oxidative stress as a consequence of Gb3 accumulation [94]. Particularly, methionine level was decreased while its oxidized form, methionine sulfoxide level increased in Fabry individuals compared to control.

This work exhibits some limitations. The current CRISPR/Cas9-modified podocyte model only enabled the exploration of the pathological mechanism of Fabry disease at the gene level. Confirmatory studies are needed to project the observed gene-expression profiles to downstream metabolic remodeling. Indeed, the regulation of the differentially expressed genes should be validated at the protein level in subsequent studies. Moreover, the *in vitro* model contains several shortcomings when it comes to *in vivo* translation. Further investigations into the resulting pathways and their interplay may unravel new therapeutic targets or strategies for modulating cellular metabolism. This may open new avenues for targeted interventions, aiming to alleviate the burden of this condition and improve patient outcomes.

## Funding sources

No specific funding.

## Data availability

Data associated with this study has not been deposited into a publicly available repository. All the data that support the findings are presented in the manuscript and the supplementary material.

## CRediT authorship contribution statement

**Sarah Snanoudj:** Writing – review & editing, Writing – original draft, Methodology, Investigation, Formal analysis, Data curation. **Céline Derambure:** Writing – review & editing, Methodology, Data curation. **Cheng Zhang:** Writing – review & editing, Methodology, Data curation. **Nguyen Thi Hai Yen:** Writing – review & editing, Writing – original draft, Methodology, Formal analysis. **Céline Lesueur:** Writing – review & editing, Methodology, Data curation. **Sophie Coutant:** Writing – review & editing, Methodology, Data curation. **Lénaïg Abily-Donval:** Writing – review & editing, Investigation, Formal analysis, Data curation. **Stéphane Marret:** Writing – review & editing, Supervision, Investigation. **Hong Yang:** Writing – review & editing, Methodology, Formal analysis, Data curation. **Adil Mardinoglu:** Writing – review & editing, Supervision, Resources, Methodology, Investigation. **Soumeya Bekri:** Writing – review & editing, Writing – original draft, Supervision, Resources, Methodology, Investigation, Conceptualization. **Abdellah Tebani:** Writing – review & editing, Writing – original draft, Visualization, Supervision, Resources, Methodology, Investigation, Formal analysis, Conceptualization.

## Declaration of competing interest

The authors declare that they have no known competing financial interests or personal relationships that could have appeared to influence the work reported in this paper.
