# Supplementary material for: Genome-wide expression analysis in a Fabry disease human podocyte cell line
Source: Heliyon. 2024 Jul 9;10(14):e34357. doi: 10.1016/j.heliyon.2024.e34357 (PMC11295972; doi:10.1016/j.heliyon.2024.e34357)
Supplement: Multimedia component 2 [file mmc2.docx]

**Genome-wide expression analysis in a Fabry disease human podocyte cell line**

Sarah Snanoudj^1^, Céline Derambure^2^, Cheng Zhang^3^, Nguyen Thi Hai Yen^1^, Céline Lesueur^1^, Sophie Coutant^2^, Lénaïg Abily-Donval^4^, Stéphane Marret^4^, Hong Yang^3^, Adil Mardinoglu^3, 5^, Soumeya Bekri^1^, Abdellah Tebani^1^*

**Affiliations**

^1^ Normandie Univ, UNIROUEN, INSERM U1245, CHU Rouen, Department of Metabolic Biochemistry, Referral Center for Lysosomal Diseases, Filière G2M, 76000 Rouen, France; [sarah.snanoudj@gmail.com](mailto:sarah.snanoudj@gmail.com) (S.S.); [celine.lesueur@chu-rouen.fr](mailto:celine.lesueur@chu-rouen.fr) (C.L.); [thi-hai-yen.nguyen@univ-rouen.fr](mailto:thi-hai-yen.nguyen@univ-rouen.fr) (N.T.H.Y.); [abdellah.tebani@chu-rouen.fr](mailto:abdellah.tebani@chu-rouen.fr) (A.T.); [soumeya.bekri@chu-rouen.fr](mailto:soumeya.bekri@chu-rouen.fr) (S.B.)

^2^ Normandie Univ, UNIROUEN, INSERM U1245 and CHU Rouen, Department of Genetics and reference center for developmental disorders, FHU-G4 Génomique, F-76000, Rouen, France; celine.derambure1@univ-rouen.fr (C.D.); [s.coutant@chu-rouen.fr](mailto:s.coutant@chu-rouen.fr) (S.C.)

^3^ Science for Life Laboratory, KTH - Royal Institute of Technology, Stockholm, Sweden; [adilm@scilifelab.se](mailto:adilm@scilifelab.se) (A.M.); [cheng.zhang@scilifelab.se](mailto:cheng.zhang@scilifelab.se) (C.Z.); [hong.yang@scilifelab.se](mailto:hong.yang@scilifelab.se) (H.Y.)

^4^ Normandie Univ, UNIROUEN, INSERM U1245, CHU Rouen, Department of Neonatal Pediatrics, Intensive Care, and Neuropediatrics, 76000 Rouen, France; [lenaig.donval@gmail.com](mailto:lenaig.donval@gmail.com) (L.A.D.); [stephane.marret@chu-rouen.fr](mailto:stephane.marret@chu-rouen.fr) (S.M.)

^5^ Centre for Host-Microbiome Interactions, Faculty of Dentistry, Oral & Craniofacial Sciences, King's College London, London, United Kingdom; [adilm@scilifelab.se](mailto:adilm@scilifelab.se) (A.M.)

***** Correspondence

Assoc. Prof. Abdellah TEBANI

Department of Metabolic Biochemistry,

Rouen University Hospital

76000 Rouen Cedex

France

[abdellah.tebani@chu-rouen.fr](mailto:abdellah.tebani@chu-rouen.fr)


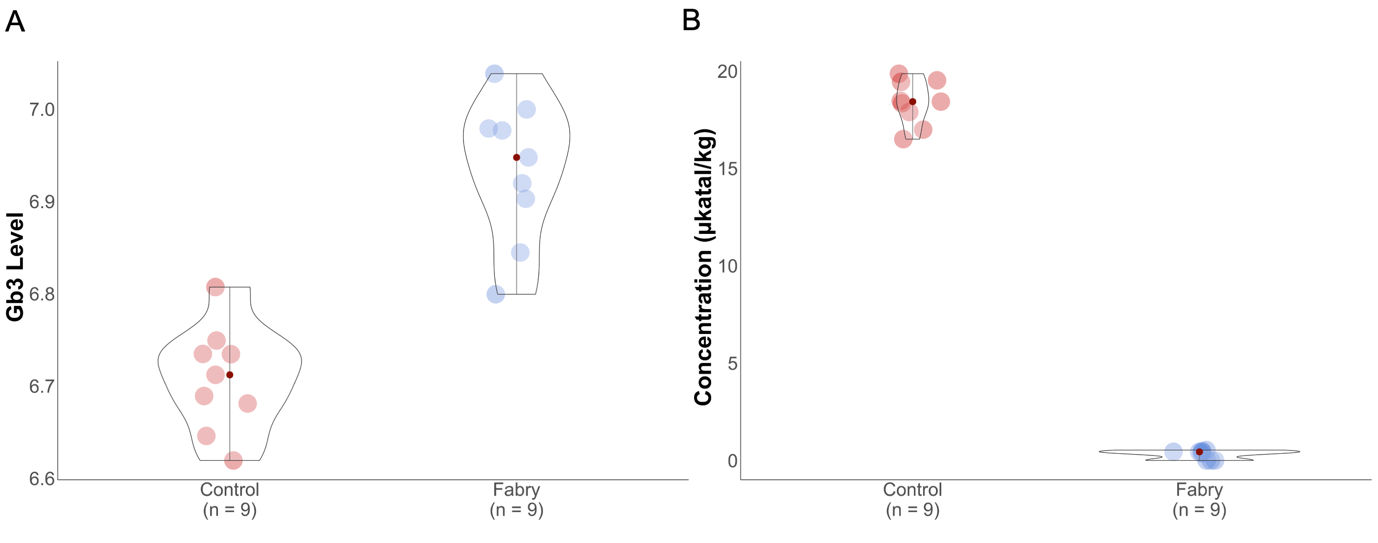


**Supplementary Figure 1.** **CRISPR/Cas9-modified podocyte model validation.** A) Gb3 accumulation in GLA-edited and control podocytes. B) α-gal A enzyme activity in GLA-edited and control podocytes. Fabry, GLA-edited podocytes; Control, control podocytes.
